# Thermal expansion of LaB_6_ from 298 to 998 K

**DOI:** 10.1107/S1600576725005898

**Published:** 2025-08-13

**Authors:** E. Koray Akdoğan, E. Andrew Payzant

**Affiliations:** a Materials Science and Engineering Department, Rutgers University, Piscataway, New Jersey08904, USA; b Neutron Scattering Division Oak Ridge National Laboratory Oak Ridge Tennessee37830 USA; Institut de Recherche sur les Céramiques, France

**Keywords:** thermal expansion, temperature calibration, high-temperature X-ray diffractometry, LaB_6_ standard material

## Abstract

We propose LaB_6_ as a temperature calibration standard for high-temperature (HT) X-ray diffractometry owing to its high temperature stability. Such HT applications require a reliable HT lattice parameter or, equivalently, peak position data, which have not been readily accessible to the diffraction community to date. As such, the thermal expansion behavior of NIST SRM 660a LaB_6_ was assessed in the temperature range 298–998 K using HT Bragg–Brentano parafocusing θ:θ X-ray diffractometry in conjunction with Rietveld analysis. Data were collected in the 2θ range 20–150° at a data collection rate of 0.5° θ min^−1^ in air and at 1 atm. The temperature was stepped in 50 K increments. The cubic unit-cell lattice parameter [*a*(*T*)] of LaB_6_ in Å was found to vary as *a*(*T*) = 4.15678 (±0.00001) + Ξ(*T* − 298 K) + Ψ(*T* − 298 K)^2^, where Ξ = 2.4645 × 10^−5^ (±4.8904 × 10^−8^) Å K^−1^ and Ψ = 1.0325 × 10^−8^ (±6.7376 × 10^−11^) Å K^−1^. The isobaric volume thermal expansion coefficient (TEC) was obtained as α_V_^P^  = (5.9291 × 10^−6^) + (4.9680 × 10^−9^)(*T* − 298 K) K^−1^, from which the corresponding linear TEC was obtained as α_L_^P^ = (1.9764 × 10^−6^) + (1.6560 × 10^−9^)(*T* − 298 K) K^−1^. The 3 × 3 matrix representations of the single-crystal isobaric linear TEC and the volume expansivity were obtained for the cubic crystal class to which LaB_6_ belongs. Also, the temperature dependence of the lattice parameter data of this study was compared with past landmark studies on LaB_6_ by Dutchak *et al.* [*Inorg. Mater.* (1972), **8**, 1877–1880] and Aivazov *et al.* [*Inorg. Mater.* (1979), **15**, 1015–1016].

## Abstract 2

The isobaric thermal expansion coefficient of LaB_6_ at 1 atm was assessed from 298 to 998 K. We propose it as a temperature calibration standard for high-temperature X-ray diffraction work owing to its high temperature stability.

## 1. Introduction

Thermal expansion is an anharmonic property of solids which originates from the asymmetry of interatomic potentials between the constituent atoms or molecules (Leibfried & Ludwig, 1961 ▸; Born & Huang, 1954 ▸; Cowley, 1968 ▸). Therefore, the assessment of the temperature dependence of thermal expansion and related properties such as compressibility, volume expansivity and temperature dependence of density, among others, is of paramount importance to gain insight into the constitution of a given solid at the level of the chemical bond and the associated thermal degrees of freedom (Born & Huang, 1954 ▸; Fultz, 2010 ▸).

On the other hand, thermal expansion causes what is known as stress-free strain under a given temperature difference (Δ*T*), especially in crystalline solids, leading to phenomena of crucial technological importance such as delamination in thin films and coatings from substrates, strain-dependent dielectric behavior of epitaxial thin films, deformation of electrochemical cells, buckling of structural metallic components, and fracture of multiphase materials (Yu & Hutchinson, 2003 ▸; Kim *et al.*, 2006 ▸; Kim *et al.*, 2007 ▸; Kim *et al.*, 2022 ▸; Evans & Hutchinson, 1995 ▸; Paxton *et al.*, 2015 ▸; Simon *et al.*, 2006 ▸; Rice, 1990 ▸; Biçer *et al.*, 2020 ▸; Biçer *et al.*, 2022 ▸). Therefore, the characterization of materials by X-ray diffraction (XRD hereafter) as a function of temperature is germane to scientific as well as technological endeavors.

Besides measuring thermal expansion in the context of the aforementioned phenomena, *in situ* lattice parameter measurements of crystalline solids as a function of temperature are also of the utmost importance in the study of crystalline defect chemistry (Krivoglaz, 1969 ▸; Kobayashi *et al.*, 2023 ▸), phase transformations (Akdoğan *et al.*, 2005 ▸; Akdoğan & Safari, 2002 ▸; Akdoğan & Safari, 2007 ▸; Aytürk *et al.*, 2008 ▸;Simos *et al.*, 2017 ▸; Ude *et al.*, 2014 ▸; Tsuji *et al.*, 2010 ▸; Haun *et al.*, 1987 ▸), solubility limits in the context of phase equilibria (Garmroudi *et al.*, 2021 ▸) and strains (Xiong *et al.*, 2019 ▸), to name but a few. In such high-temperature measurements, one typically uses a resistively heated hot stage (Fantner *et al.*, 1998 ▸; Fischer & Lersch, 1998 ▸). The temperature is concomitantly measured with a thermocouple that is typically spot welded to the underside of the hot stage, which is subjected to Joule heating (Fantner *et al.*, 1998 ▸; Fischer & Lersch, 1998 ▸). In this configuration, temperature gradients as large as tens of degrees across the hot stage and specimen have been reported, which poses major problems for high-temperature X-ray diffractometry (HTXRD hereafter) in terms of accuracy (Fischer & Lersch, 1998 ▸; Beck & Mittemeijer, 2002 ▸). Thermocouple measurement errors arising from voltage gradients across the heater strip are also possible (Fantner *et al.*, 1998 ▸; Fischer & Lersch, 1998 ▸).

Errors in lattice parameter measurements caused by specimen height displacement in XRD work that is based on the Bragg–Brentano parafocusing technique can be minimized by employing internal/external standards or by analytical methods in the related data analysis (King & Payzant, 1992 ▸; Beck & Mittemeijer, 2002 ▸). However, there is no well established approach to handle errors originating from inaccurate temperature measurement for *in situ* HTXRD (Pitschke & Teresiak, 1998 ▸). In principle, an internal standard will (i) not react with and (ii) not overlap the peaks of the sample of interest. There have been various landmark studies of temperature calibration in high-temperature X-ray powder diffraction that are based on the differential thermal expansion method (Drews, 2001 ▸) and speciality high-temperature chambers (Dapiaggi *et al.*, 2002 ▸), among others. Also, high-purity platinum powder has been used as a high-temperature standard (Kirby, 1991 ▸). In principle, the temperature calibration should be done by the use of a thermal expansion standard that is easy to deploy, that is readily available, and whose effectiveness is not dependent on the hot stage or furnace being used. As such, it is valuable to have a selection of thermal expansion powder standards that can be matched with a given sample. We therefore propose herein to use lanthanum hexaboride (LaB_6_) as a temperature standard to surmount the aforementioned challenges in HTXRD. Accordingly, the data provided for LaB_6_ in this study have substantial potential for implementation in a broad range of materials research involving HTXRD.

**Figure 1:**
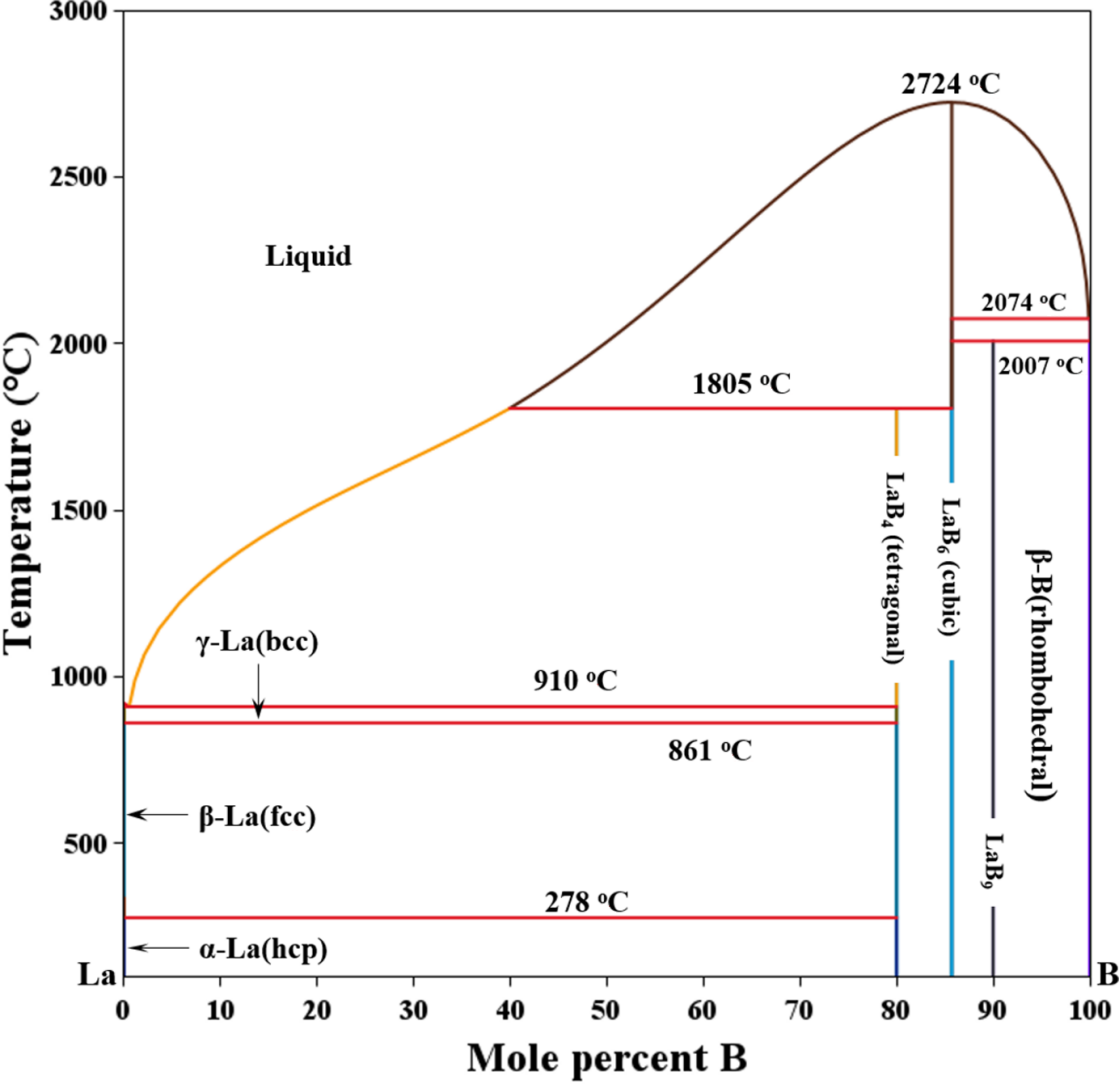
The La–B binary phase diagram as computed by *Thermo-Calc*.

As shown on the phase diagram in Fig. 1 ▸, which was computed with *Thermo-Calc* (Andersson *et al.*, 2002 ▸) using the most up-to-date thermodynamics data, LaB_6_ is a line compound in the La–B binary system (Schlesinger *et al.*, 1999 ▸). While the normal melting temperature of LaB_6_ is reported as 2210°C (2483 K) in some prior references (Lide, 1993 ▸), it is in fact a compound that melts incongruently at 2724°C (see Fig. 1 ▸). It has no phase transformations for *T* < 1805°C (see Fig. 1 ▸), providing a wide and steady working temperature range (Takahashi *et al.*, 1999 ▸). Moreover, LaB_6_ possesses good chemical stability and oxidation resistance (Gogotsi *et al.*, 1987 ▸), making it a good reference material for HTXRD. Specifically, LaB_6_ has an oxidation onset temperature of 670–700°C (Gogotsi *et al.*, 1987 ▸). This oxidation is characterized by the formation of a thin protective oxide scale at temperatures up to about 1200°C (Lavrenko *et al.*, 1973 ▸). Hence, LaB_6_ should be very suitable as either an internal or an external standard for calibration of temperature in high-temperature diffractometry up to at least 670°C in the presence of oxygen and to much higher temperatures in inert atmospheres. Such applications require reliable high-temperature lattice parameter data (or, equivalently, peak positions), which have not been readily available to the X-ray diffraction community to date. We also note that LaB_6_ crystallizes in the cubic crystal class, *i.e.Pm*3*m*. Therefore, LaB_6_ ideally lends itself to structure refinement since one can obtain high-quality lattice parameter data thanks to its regular, well spaced, sharp diffraction peaks over a wide range of *d*-spacing values. Furthermore, phase-pure LaB_6_ is readily available and can be sourced from many vendors if certification by the National Institute of Standards and Technology (NIST hereafter) is not required. Otherwise, LaB_6_ can be acquired as a standard reference material (SRM 660a–c) for line broadening and peak position calibration from NIST as well (Cline *et al.*, 2000 ▸). Hence, LaB_6_ can be reliably obtained and used for high-temperature work.

In what follows, we present thermal expansion data for LaB_6_ NIST SRM 660a which will enable its implementation as an internal or external temperature standard for HTXRD. We thereby hope to contribute to establishing a standardized error-handling protocol for high-temperature diffractometry.

## 2. Experimental and data analysis

LaB_6_ powder that was certified by NIST as SRM 660a (see https://www.nist.gov/srm) was used in this study. The average particle size of SRM 660a was ∼9 µm with a range of 4–15 µm (Cline *et al.*, 2000 ▸), which ensured the absence of line broadening arising from finite crystal size (also known as coherently diffracting domain size) (Warren, 1990 ▸; Krivoglaz, 1969 ▸) or *d*-spacing variation (also known as microstrain) (Warren, 1990 ▸; Klug & Alexander, 1974 ▸) in the collected X-ray pattern.

Data were collected using a Philips X’Pert Pro MPD θ:θ X-ray diffractometer which was configured with a curved multilayer incident beam mirror and diffracted beam ‘thin film’ parallel-plate collimator. Such a configuration ensured parallel beam optics, thus minimizing sensitivity to sample displacement errors in measuring the lattice parameter. Both incident and diffracted beam Soller slits were inserted to reduce axial divergence errors. The LaB_6_ powder was packed in the MACOR sample holder of an Anton Paar XRK900 diffractometer furnace, which was placed in a stagnant air atmosphere. The sample holder was rotated during the data collection to improve counting statistics. Data were collected over the 2θ range 20–150° at a data collection rate of 0.5° θ min^−1^.

The temperature was stepped in 50°C increments and was measured by using a NiCr/NiAl (type K) thermocouple that was in physical contact with the MACOR (glass ceramic) sample stage. The temperature of the thermocouple was recorded to a precision of 0.1°C and the accuracy was ±2°C. The difference between the thermocouple and powder sample was negligible when working in a 1 atm gas environment, even though the temperature of the powder specimen was not directly measured but inferred from the thermocouple reading. The sample displacement variation over the temperature range was found to be insignificant, *i.e.* within the error of the correction.

Diffraction data were analyzed by the Rietveld method (Rietveld, 1969 ▸) using the program *GSAS* (Larson & Von Dreele, 1985 ▸; Toby & Von Dreele, 2013 ▸). The background was defined by a third-order polynomial in 2θ and was refined simultaneously with the other variables. A Voigt profile shape function was chosen to model the line shape of the Bragg peaks (Hölzer *et al.*, 1997 ▸; Young & Wiles, 1982 ▸). The Gaussian component of the full width at half-maximum (FWHM) (Γ_G_) of the peaks was modeled according to the Caglioti equation (Caglioti *et al.*, 1958 ▸):
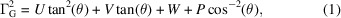
where *U*, *V* and *W* describe instrumental broadening and *P* is the Scherrer coefficient for Gaussian broadening (Scherrer, 1918 ▸). The Lorentzian component (Lutterotti & Scardi, 1990 ▸) of the FWHM width (Γ_L_) of the peaks was modeled according to
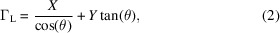
where *X* is the Scherrer broadening (Scherrer, 1918 ▸) and *Y* is the strain broadening (Lutterotti & Scardi, 1990 ▸; Balzar, 1992 ▸).

The data collected at 25°C were used to determine the appropriate Caglioti coefficients *U*, *V* and *W* for the diffractometer in question, which were found to be 0, −5.3 and +5.7, respectively. These values are reasonable given the lower resolution (*i.e.* larger FWHM) resulting from the use of a parallel-plate collimator on the diffracted beam. The polarization of the beam by the mirror was very low (*i.e.* POLA = 0.54), and the 2θ zero error was −0.01° (*i.e.* ZERO = −1). The powder data statistics were *R*_wp_ = 12.5% and *R*_p_ = 9.6%, and the goodness of fit was given by χ^2^ = 2.57.

## 3. Theoretical considerations

The thermal expansion coefficient reported in this study follows from the generalized equation of state describing the temperature and pressure dependence of volume for an arbitrary solid, as expressed by the following Pfaffian (Callen, 1960 ▸):
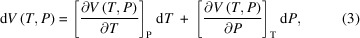
where the partial derivatives in equation (3) are related to the isobaric volume thermal expansion coefficient (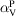) and the isothermal volumetric compressibility (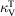) via (Callen, 1960 ▸)
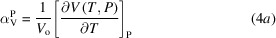
and
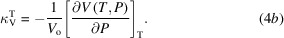
Here, *V*_o_ is the molar volume of the specimen of interest at the reference temperature θ, which is traditionally taken as 298 K. Upon substituting equations (4*a*)–(4*b*), equation (3) can be recast as
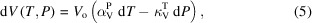
where 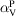 and 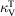 are both functions of temperature and pressure in general (Swalin, 1972 ▸). These two constitutive properties are related to each other by the Maxwell relation (Swalin, 1972 ▸),
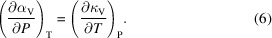
Moreover, the isobaric linear thermal expansion coefficient (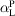) is related to its volumetric counterpart (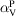) simply by 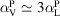 as per the binomial theorem (Arfken *et al.*, 2012 ▸) since the changes in dimension due to a temperature variation (Δ*T*) are on the order of 10^−5^ to 10^−6^ K^−1^ (Taylor, 1998 ▸).

In this study, the cubic lattice parameter of LaB_6_ was modeled by using a Taylor series expansion of the cubic lattice parameter *a* around the reference temperature θ as
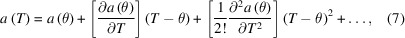
from which the isobaric linear thermal expansion was obtained according to
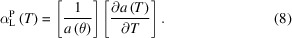
As per equation (8) in conjunction with equation (7), the temperature dependence of thermal expansion then becomes
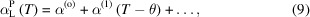
where
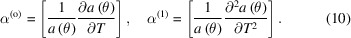


The thermal expansion coefficient constitutes a tensor of rank 2 since it relates the stress-free strain (*u_ij_*) to a change in temperature (Δ*T*), which are tensors of rank 2 and 0 (a scalar), respectively (Nye, 1985 ▸; Newnham, 2005 ▸). Hence, 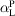 is represented by a 3 × 3 matrix (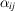) for which 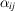
 = 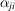 (Newnham, 2005 ▸) for *i* ≠ *j*, and where *i*, *j* = 1, 2, 3 as per the Einstein suffix notation (Nye, 1985 ▸). LaB_6_ belongs to the cubic crystal class (*m*3*m*) (see PDF Nos. 00-034-0427, 00-059-0322 or 04-003-6661; https://www.icdd.com/), which reduces the number of independent components of the isobaric linear thermal expansion tensor to one, as shown below (Nye, 1985 ▸):
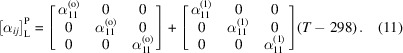
Polycrystalline solids with equiaxed grains, which belong to Curie group ∞∞*m* (Newnham, 2005 ▸), do not exhibit preferred orientation, *i.e.* no texture. Therefore, the magnitude of the thermal expansion coefficient in an arbitrary direction (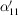) in Cartesian coordinates is given by (Newnham, 2005 ▸)
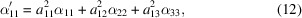
where *a*_11_, *a*_12_ and *a*_13_ are the relevant direction cosines. The average of 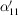 (represented as 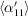 in what follows) is
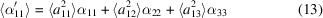
with 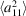 = 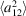 = 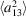 = 1/3, leading to (Newnham, 2005 ▸)
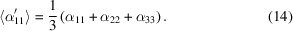
In crystalline solids belonging to cubic crystal classes such as LaB_6_, one has 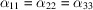, resulting in 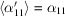. Therefore, equation (11) also represents the linear thermal expansion of polycrystalline LaB_6_ in which equiaxed grains are randomly oriented.

The volume expansivity is defined as (Newnham, 2005 ▸)
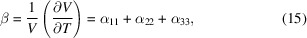
which reduces to 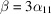 for the cubic crystal class because 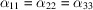 as indicated before. Hence, 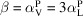, which leads to
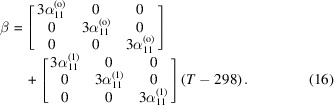
The temperature dependence of density ρ, on the other hand, is simply related to the volume expansivity β as (Newnham, 2005 ▸)
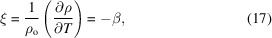
which we include here for completeness. Next, we will present the temperature dependence of the cubic cell constant for NIST SRM 660 LaB_6_ and obtain the numerical values of 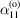, 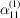, 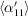, 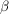 and 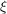.

## 4. Experimental results and discussion

**Figure 2:**
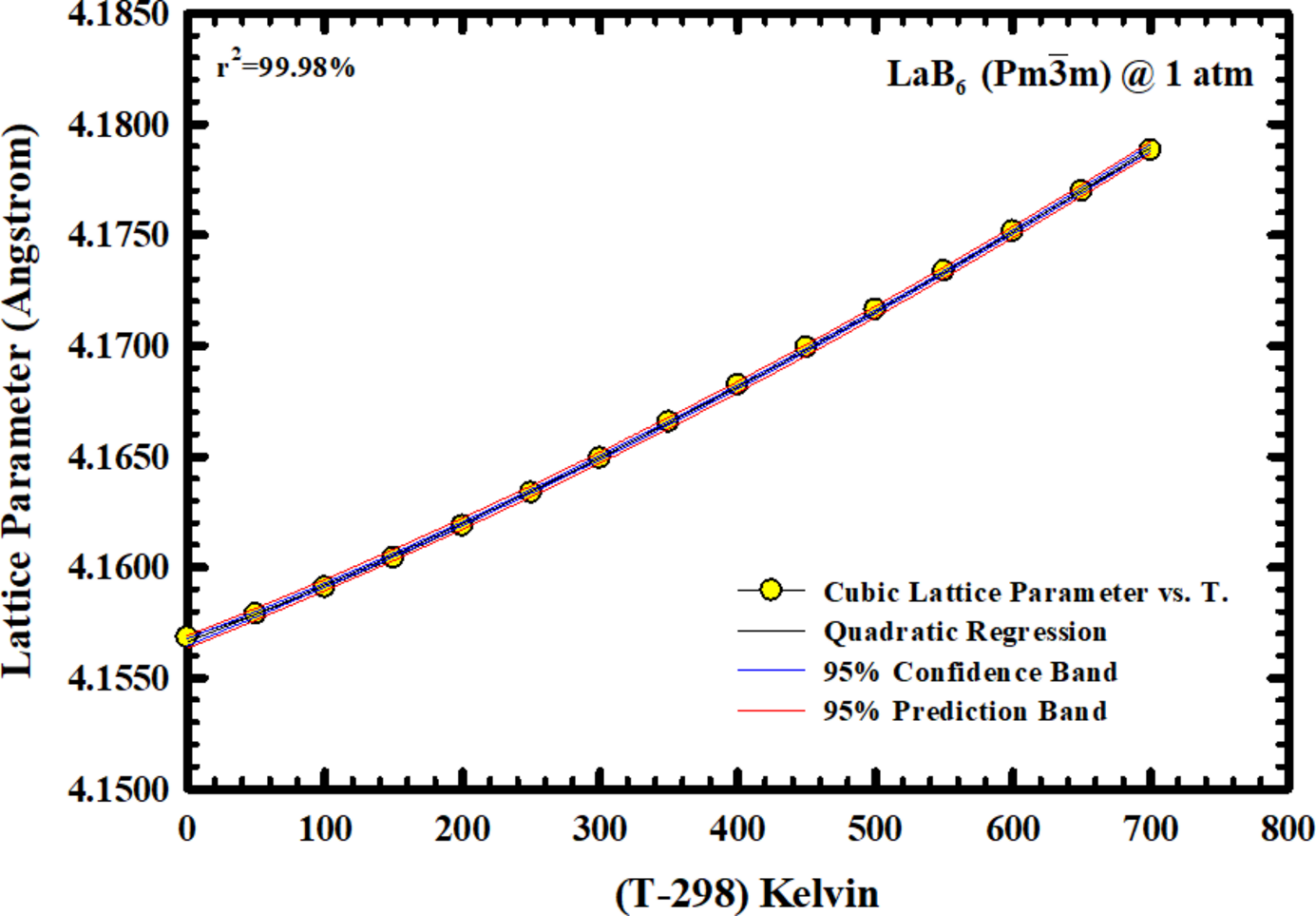
The variation of the cubic lattice parameter of LaB_6_ NIST SRM 660a from 298 to 998 K in air and at 1 atm, as determined in this study.

Fig. 2 ▸ depicts the variation of the LaB_6_ cubic lattice parameter with temperature in air over the 298–998 K range. There is a discernible, albeit small, nonlinear variation with temperature. Our initial attempt with linear regression (not shown) resulted in *r*^2^ = 99.6% and a 298 K cubic cell constant of 4.15583 (±0.00002) Å. A parabolic regression to the data (see Fig. 2 ▸) resulted in *r*^2^ = 99.98% and a 298 K cubic cell constant of 4.15678 (±0.00001) Å. The lattice parameter at 298 K that was obtained using the said parabolic regression is closer to the data reported for NIST SRM 660a, *i.e.* 4.15692 (±0.00001) Å (Cline *et al.*, 2000 ▸), than the value obtained by the linear regression. The results of the aforementioned parabolic regression can be expressed in analytical form as
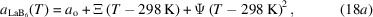
where
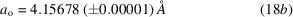

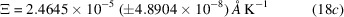

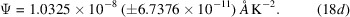


**Figure 3:**
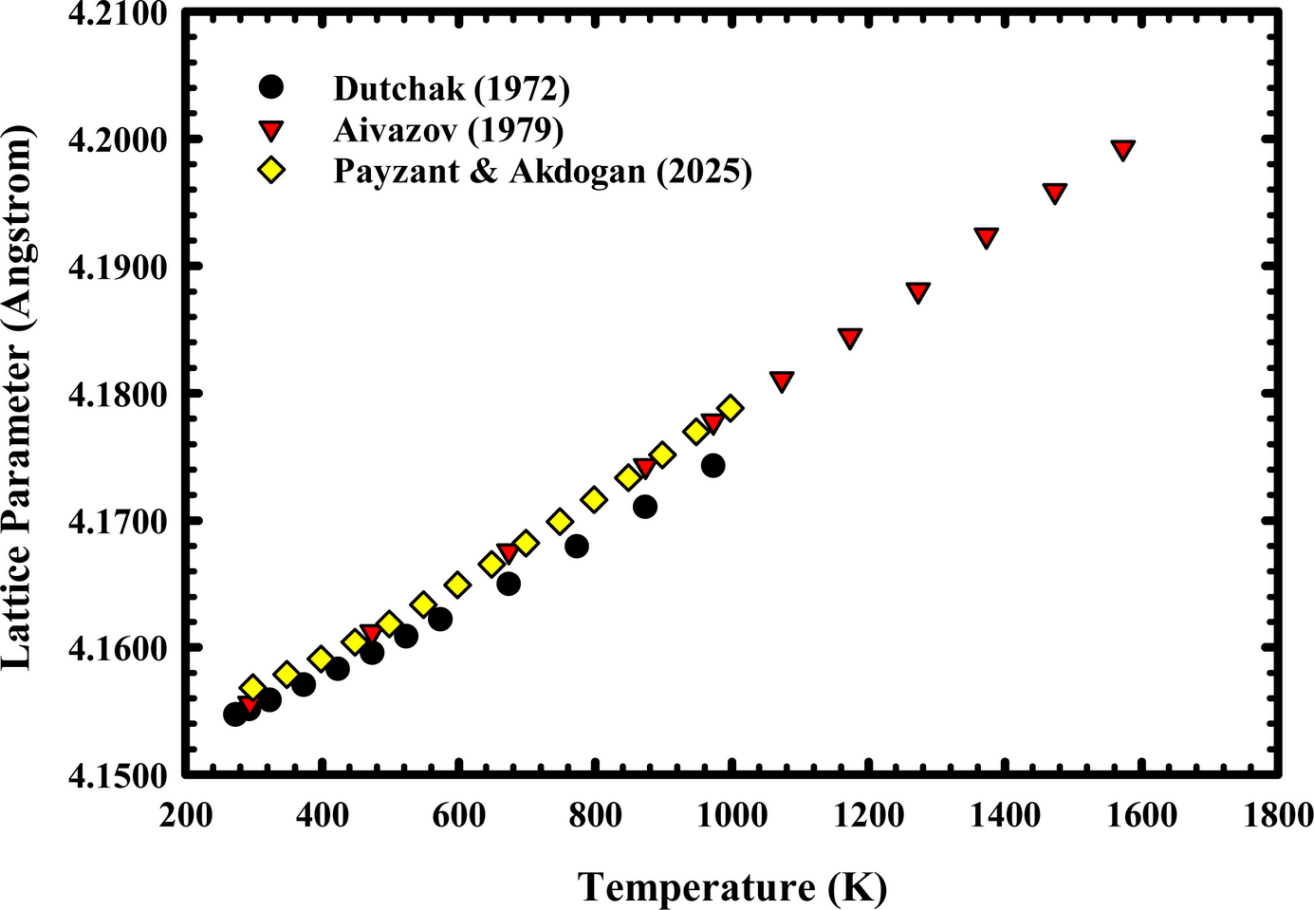
Comparison of the temperature dependence of the LaB_6_ NIST SRM 660a cubic lattice parameter obtained in this study over the range 298–998 K in air at 1 atm with that of Dutchak *et al.* (1972 ▸, 1975 ▸) and Aivazov *et al.* (1979 ▸).

As shown in Fig. 3 ▸, our data align very well with those of Aivazov *et al.* (1979 ▸) and less well with those of Dutchak *et al.* (1972 ▸, 1975 ▸). Most importantly, our data provide a better quantitative description of the temperature dependence of the LaB_6_ lattice parameter in the 298–998 K interval because of higher data density than in the work of Dutchak *et al.* (1972 ▸, 1975 ▸).

**Figure 4:**
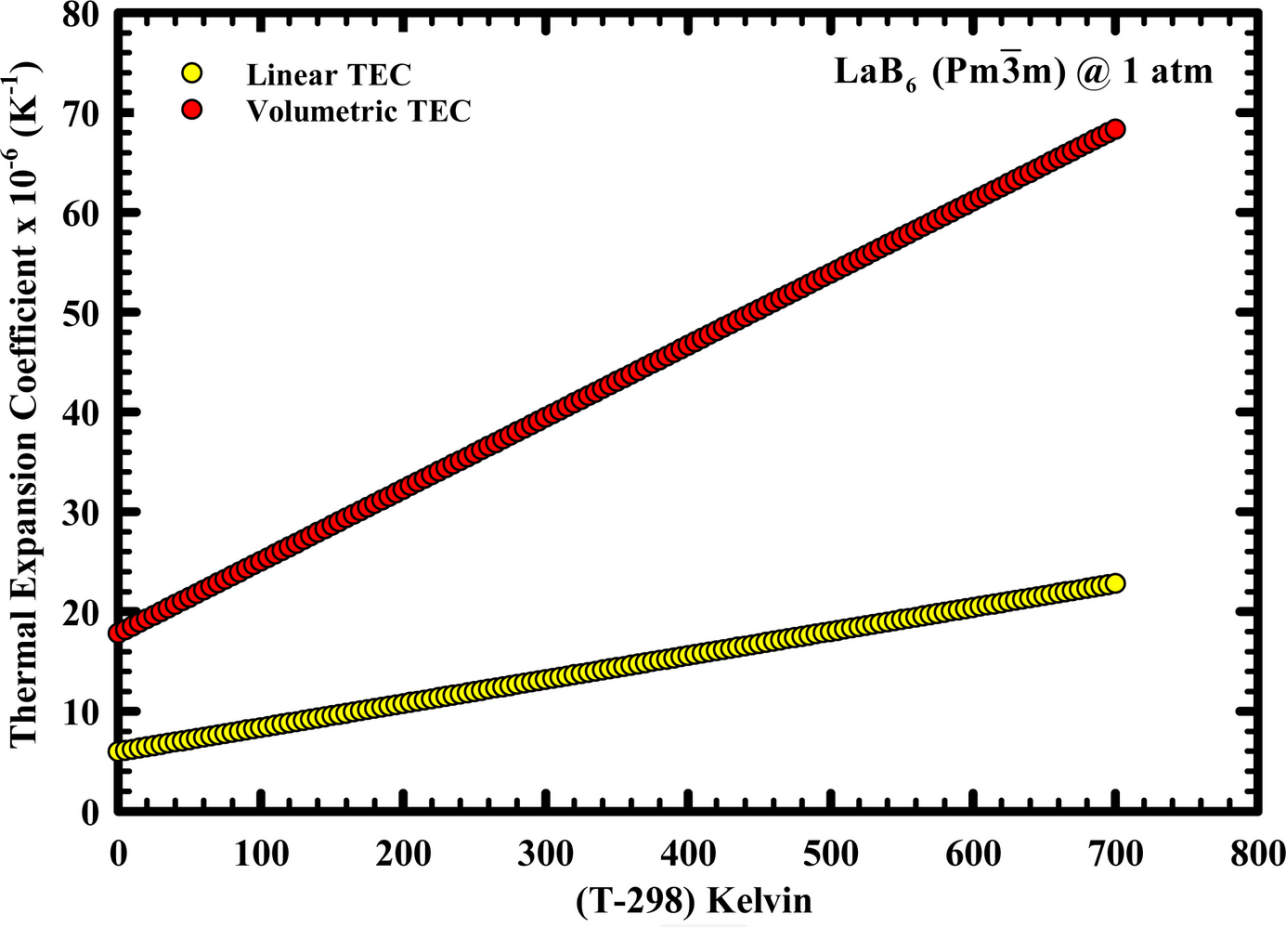
The temperature dependence of the isobaric linear and volumetric thermal expansion coefficients of LaB_6_ NIST SRM 660a, as obtained in this study from 298 to 998 K in air at 1 atm.

Fig. 4 ▸ shows the variation of the isobaric linear and the volumetric thermal expansion coefficients of LaB_6_ over the interval 298–998 K, obtained via equations (6)–(8). According to the data in hand, the temperature dependence of the isobaric volumetric thermal expansion coefficient can be expressed phenomenologically as follows:
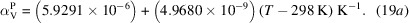
The variation of the thermal expansion coefficient is linear. Modeling of the lattice parameter with a cubic polynomial did not lead to a meaningful *T*^3^ dependence of the lattice parameter in our preliminary analysis which is why we did not implement it, *i.e.* the cubic dependence was physically insignificant. The *r*^2^ of the parabolic fit is 99.98%, which we considered as satisfactory. We attribute the linearity to the fact that the temperature over which our measurement was carried out is approximately ∼25% of the normal melting temperature of LaB_6_, *i.e.* 2724°C. In most materials, the thermal expansion coefficient exhibits nonlinearity when the temperature is typically above ∼0.6 of the melting temperature (Callen, 1960 ▸; Newnham, 2005 ▸). The magnitude of such nonlinearity, which arises from the anharmonic characteristics of interatomic potentials, becomes pronounced as the melting temperature is approached (Born & Huang, 1954 ▸; Cowley, 1968 ▸).

The isobaric linear thermal expansion is also depicted in Fig. 4 ▸, which was obtained from the binomial theorem (see Section 3). Its corresponding phenomenological representation is
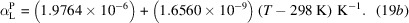


The single-crystal isobaric linear thermal expansion matrix for LaB_6_ follows from equation (9) in conjunction with equation (19*b*) as
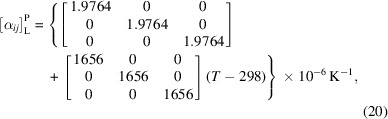
which also applies to LaB_6_ in the equiaxed (isotropic, Curie group ∞∞*m*; Newnham, 2005 ▸) polycrystalline state of aggregation according to equation (13). On the other hand, the variation of the volume expansivity with temperature for LaB_6_ follows from equation (15) in conjunction with equation (19*b*) as
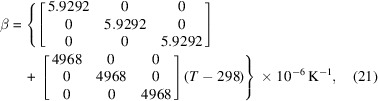
from which the temperature dependence of density can be obtained as −β which we will not reproduce here for brevity.

The approach and the results presented herein could effectively be implemented in high-temperature energy-dispersive synchrotron XRD (Croft *et al.*, 2009 ▸) work, which is a transmission method where the sample is stationary. The approach reported herein can also be extended to high-temperature neutron diffraction (Nycz *et al.*, 2021 ▸) work with some precautions. The ^10^B isotope of boron has a very high neutron absorption cross section (∼3840 barns for thermal neutrons), whereas that for the ^11^B isotope is only ∼0.005 barns (Carter *et al.*, 1953 ▸). Since natural boron contains ∼20% ^10^B and ∼80% ^11^B, non-standardized LaB_6_ will exhibit appreciable neutron attenuation, thereby affecting the data quality of the Bragg reflections (Sun *et al.*, 2023 ▸; Murthy *et al.*, 2020 ▸). While attenuation by ^10^B primarily affects peak intensities due to attenuation, systematic errors in peak position may occur and are typically attributed to factors such as (i) preferred absorption of certain paths (geometric bias), (ii) incorrect absorption correction in Rietveld refinement or profile fitting, and (iii) thermal gradients or sample heating. Hence, peak positions may appear shifted due to altered effective path lengths and scattering volumes if the sample has a high ^10^B content and the absorption is not corrected properly. As a result, such artifacts can indeed mimic lattice strain or thermal expansion which, of course, needs to be avoided. The use of thin specimens and standardized ^11^B-enriched LaB_6_ (Black *et al.*, 2020 ▸) material is recommended, together with proper absorption correction during data refinement.

The deployment of LaB_6_ as a temperature calibration standard in HTXRD requires some further considerations to ensure the errors associated with temperature calibration can be minimized. Therefore, the following strategies are recommended:

(i) *Errors associated with temperature measurement*. The actual temperature of the powder sample can be different from the temperature reported by the sensor in the hot stage of the diffractometer (McGuire *et al.*, 2008 ▸). As such, judicious choice of the hot stage type is important. The hot stage used in this study (Anton Paar XRK900) enables one to measure the temperature with high accuracy, requiring no correction as the error in temperature measurement is negligible. However, the authors have experience with other high-temperature and low-temperature XRD systems for which the change in displacement with temperature can be very large. For instance, these shifts are often different from run to run in metal foil (heater strip) based systems, requiring the meticulous use of an internal standard for correction.

(ii) *Sample displacement errors caused by the thermal expansion/contraction of the sample or sample stage with respect to the X-ray beam*. Such errors can be corrected using standards whose peak positions are known as a function of temperature. Specifically, standards with a large thermal expansion coefficient (TEC) are suitable for temperature correction and samples with a low TEC may be suitable for sample displacement correction. Moreover, external standards can be used to generate a correction for the instrument (independent of the sample), but this requires confidence that the same conditions can be reproduced with the sample as with the standard.

Alternatively, mixing a standard with the sample enables co-refinement of the two (or more) materials with confidence as the conditions for sample and standard are identical, but with some new complications. Firstly, one must ensure that the standard and sample are compatible over the temperature range of study, and also with the sample holder and gas environment. Secondly, the ideal standard must have well resolved peaks that are not overlapped with those of the specimen of interest. Such a strategy requires one to have a selection of standards in order to identify those that are not significantly overlapping with all the sample’s Bragg peaks of interest. Hence, it is essential to have many potential standards and it is for this reason we present this study on LaB_6_, a standard with regularly spaced sharp peaks that can be easily co-refined in mixed powder samples.

It follows from the foregoing that LaB_6_ can be deployed as a temperature calibration standard while still serving as a line broadening and peak position standard for a multitude of diffraction techniques.

## 5. Concluding remarks

LaB_6_ is a well established standard reference material that is extensively used as a line profile and peak position calibration standard in X-ray diffractometry. Owing to its high temperature stability, we proposed to use LaB_6_ as a high-temperature standard for temperature calibration, thereby widening its range of application. In high-temperature X-ray diffractometry, the accurate measurement of the sample temperature is crucial for assessing thermally driven phenomena such as crystallization and structural and diffusional phase transitions, as well as evaluation of the temperature dependence of solubility, macrostrain or *d*-spacing variations in a multitude of circumstances associated with the solid state of crystalline matter, to name but a few. The thermal expansion and related constitutive properties of LaB_6_ reported herein will enable one to calibrate the temperature data from 25 to 725°C (298–998 K) under 1 atm in air for a given hot stage that is used in HTXRD. In so doing, the temperature evolution of the aforementioned phenomena can be analyzed with great accuracy. The proposed approach, while demonstrated for Bragg–Brentano methods in this study, can be extended to other diffraction techniques such as energy-dispersive X-ray diffractometry and neutron diffractometry. For such purposes, LaB_6_ can be used as either an internal standard or an external standard.
